# Life-Threatening Haemorrhagic Enterocolitis: A Rare Complication of Chemotherapy With Cyclophosphamide

**DOI:** 10.7759/cureus.79534

**Published:** 2025-02-23

**Authors:** Gousmahammad Myageri, Ajith Kumar AK, Abhijith Bale, Yathish GC, Venkatesha Gupta KV

**Affiliations:** 1 Critical Care Medicine, Aster Whitefield Hospital, Bengaluru, IND; 2 Gastroenterology, Aster Whitefield Hospital, Bengaluru, IND; 3 Rheumatology, Aster Whitefield Hospital, Bengaluru, IND

**Keywords:** complication of treatment, cyclophosphamide therapy, haemorrhagic enterocolitis, life threatening, sjögren's syndrome

## Abstract

Cyclophosphamide (CPA) is a commonly used alkylating agent with its use extending from chemotherapy to immunosuppression. Herein, we present a case of a 61-year-old lady diagnosed with Sjögren’s syndrome one year ago, who recently started on CPA therapy and presented to ICU with the sudden onset of bloody diarrhoea and shock, requiring fluid resuscitation and vasopressor support. An extensive evaluation, including stool routine analysis, cultures, stool BioFire® FilmArray® (gastrointestinal panel) (BioFire Diagnostics, LLC, Salt Lake City, USA), contrast-enhanced CT (CECT) of the abdomen, colonoscopy, and biopsy, ruled out infectious, inflammatory, ischemic, and malignant causes. After discontinuation of CPA and supportive therapy, her clinical condition improved, and she was discharged. Haemorrhagic cystitis, myelosuppression, gonadal toxicity and interstitial pneumonia are well-known side effects of CPA. Haemorrhagic enterocolitis is a rare adverse effect of CPA therapy. Through this case report, we aim to highlight that haemorrhagic enterocolitis can be a potentially life-threatening adverse effect of CPA, a drug widely used in rheumatological and oncological conditions. Increased awareness among treating physicians can help in early suspicion and appropriate management, including prompt discontinuation of the drug and consideration of alternative treatment options.

## Introduction

Sjögren’s syndrome is an autoimmune disorder seen in middle-aged individuals with female preponderance [1,2]. It affects glandular tissue, especially the lacrimal and salivary glands. Patients may also develop extra-glandular involvement in organs such as the joints, skin, lungs, gastrointestinal (GI) tract, nervous system, and kidneys. Symptoms include dryness of the eyes and mouth. Sjögren’s syndrome can also be associated with other autoimmune disorders including rheumatoid arthritis (RA) and systemic lupus erythematosus (SLE). Treatment is directed towards replacing moisture in affected organs, administering steroids and, in refractory cases, pulse doses of cyclophosphamide (CPA) have also been tried [3,4].

CPA is an anti-neoplastic agent which acts through the alkylation of DNA. The drug is metabolized to an active compound which inhibits protein synthesis through DNA and RNA cross-linking. CPA is broken down in the liver by the enzyme cytochrome P-450, first to hydroxycyclophosphamide and then to aldophosphamide. Aldophosphamide is broken down to the active alkylating agent phosphoramide mustard and acrolein. It is this phosphoramide metabolite which forms cross-linkages within and between adjacent DNA strands. These cross-links lead to cell death [5,6].

Low doses of CPA have also been used as a part of immunosuppressive therapy as it eliminates regulatory T cells in naïve or malignant host cells, induces T cell growth factors like type I interferons and reduces alloreactivity. Common side effects include haemorrhagic cystitis, amenorrhea, myelosuppression, alopecia and gonadal suppression [7,8]. Haemorrhagic cystitis is a very well-known side effect of CPA due to the acrolein component. To the best of our knowledge, only one case of haemorrhagic enterocolitis and three cases of severe enteritis following the use of CPA have been reported in the medical literature so far [9-12]. Here, we present a rare case of life-threatening haemorrhagic enterocolitis secondary to CPA therapy.

## Case presentation

**Figure 1:**
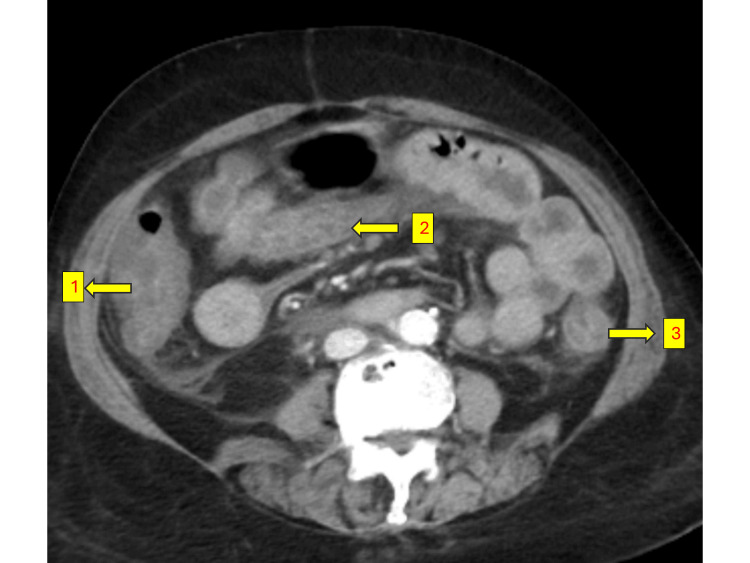
Thickened Jejunal Loops (1) Ascending colon; (2) Jejunal loops; (3) Descending colon

**Figure 2:**
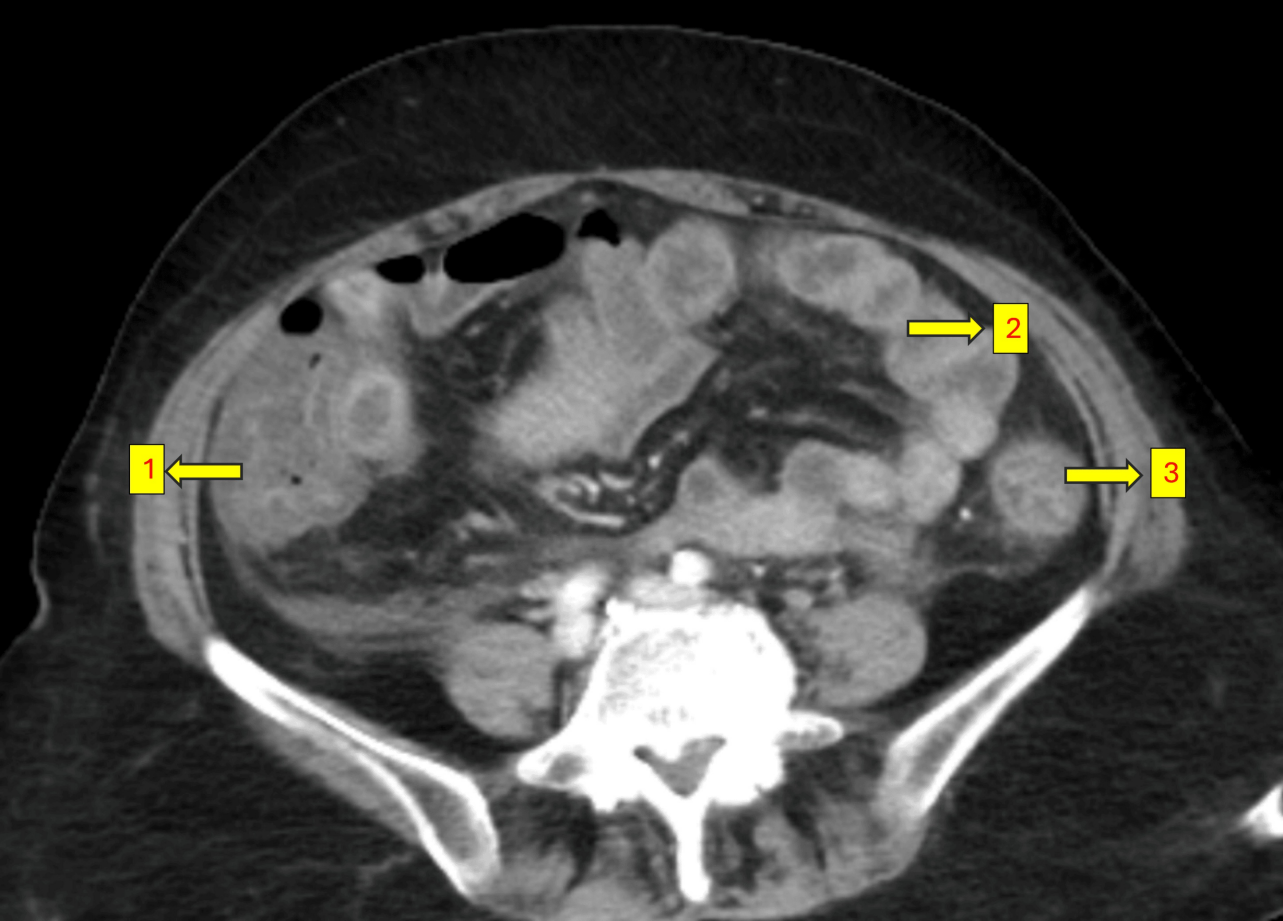
Thickened Ascending and Descending Colon With Mucosal Enhancement (1) Ascending colon; (2) Bowel loops showing mucosal enhancement; (3) Descending colon

**Figure 3:**
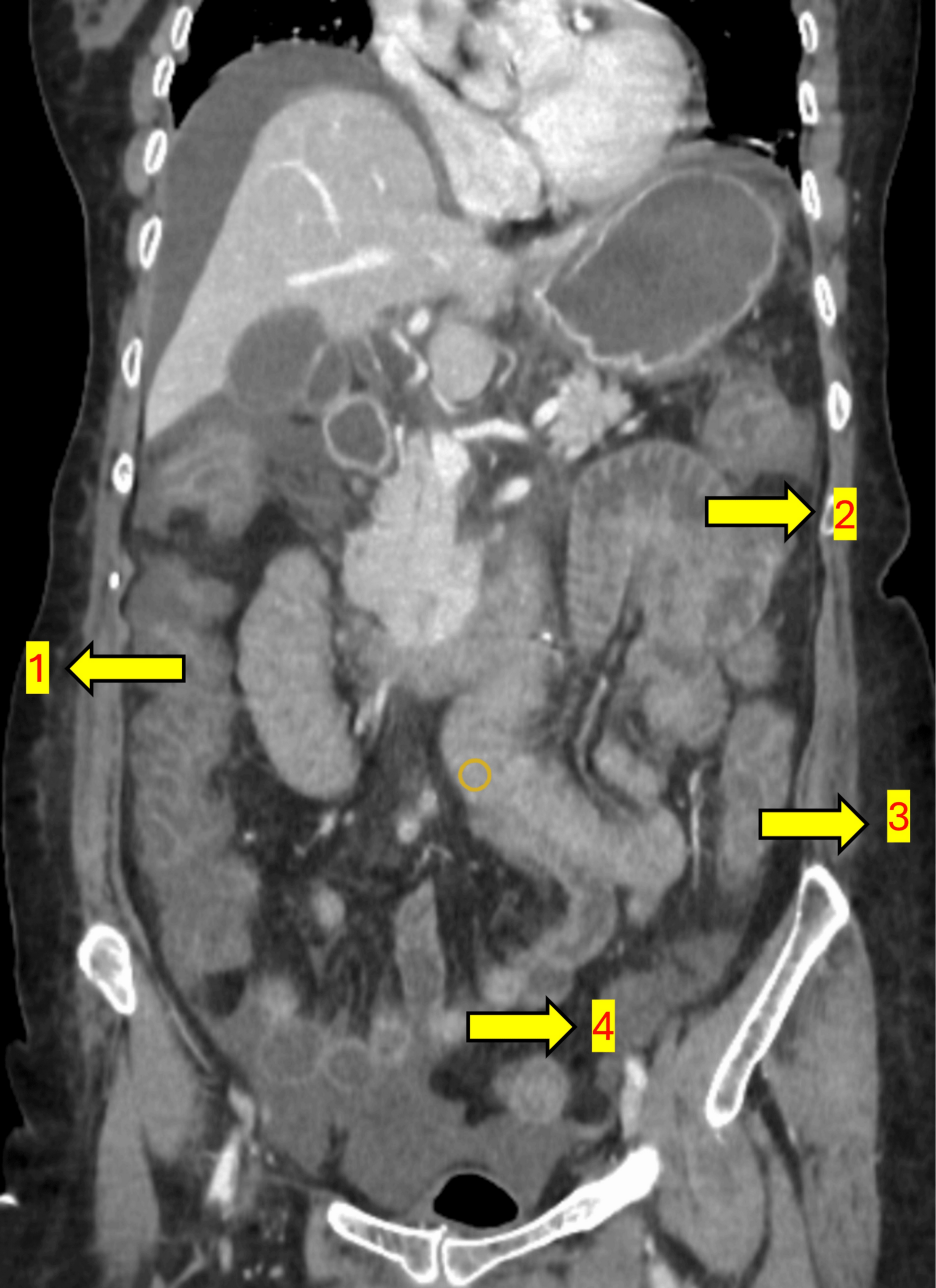
Coronal Section of the Abdomen Showing Bowel Wall Thickening (1) Thickened ascending colon; (2) Thickened jejunal loops; (3) Thickened descending colon; (4) Thickened ileal loops

A 61-year-old female with a history of diabetes, hypertension, and hypothyroidism presented with recurrent oral ulcers, dryness of the eyes and mouth, and reduced food intake for the past year. She was evaluated extensively elsewhere and found to have Sjögren’s syndrome with vasculitic ulcers. She was referred to our centre for the continuation of CPA therapy which was ongoing. She received five cycles of the CPA infusion protocol, consisting of 500 mg in 300 mL of normal saline (NS) over three hours, along with intravenous mesna (200 mg) administered before and after the CPA infusion. Two days after the fifth cycle, she presented to the emergency room (ER) with multiple episodes of hematochezia (bloody loose stools) and vomiting. She also complained of severe, diffuse pain abdomen. On arrival at the ER, she was tachycardic, hypotensive and oliguric. Blood gas analysis revealed normal anion gap metabolic acidosis. Preliminary investigations revealed creatinine of 3.6 mg/dL. Abdominal ultrasound (USG) was performed, raising suspicion of intussusception or intestinal obstruction. After obtaining consent from the family, considering the deranged renal parameters, a contrast-enhanced CT (CECT) of the abdomen was performed, which revealed diffuse thickening of the mucosal walls of the small and large intestines, with no evidence of intestinal obstruction, mesenteric ischemia, or intussusception (Figures 1-3).

A screening echocardiogram revealed a kissing left ventricle (LV) and a collapsing inferior vena cava (IVC), suggestive of severe hypovolemia. The patient was resuscitated with IV fluids, started on inotropes for persistent hypotension and subsequently shifted to the medical intensive care unit (MICU) for further management.

**Figure 4:**
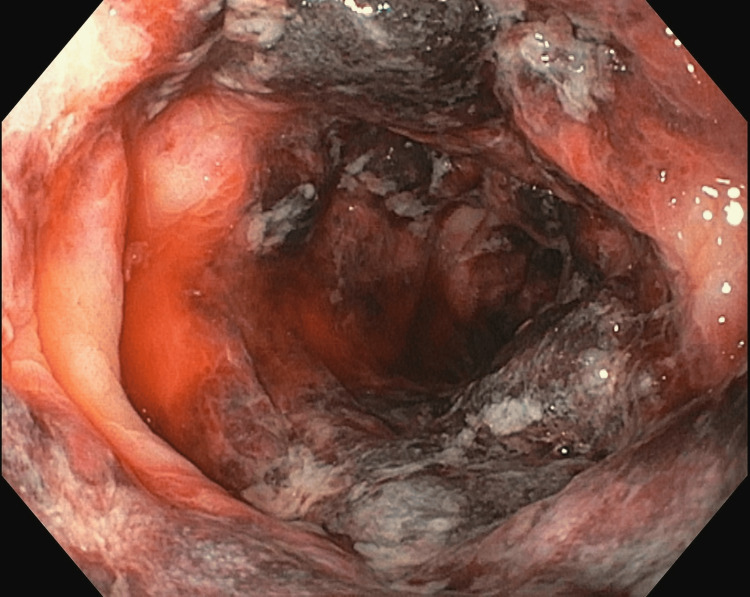
Colonoscopy Showing Mucosal Edema and Haemorrhage

**Figure 5:**
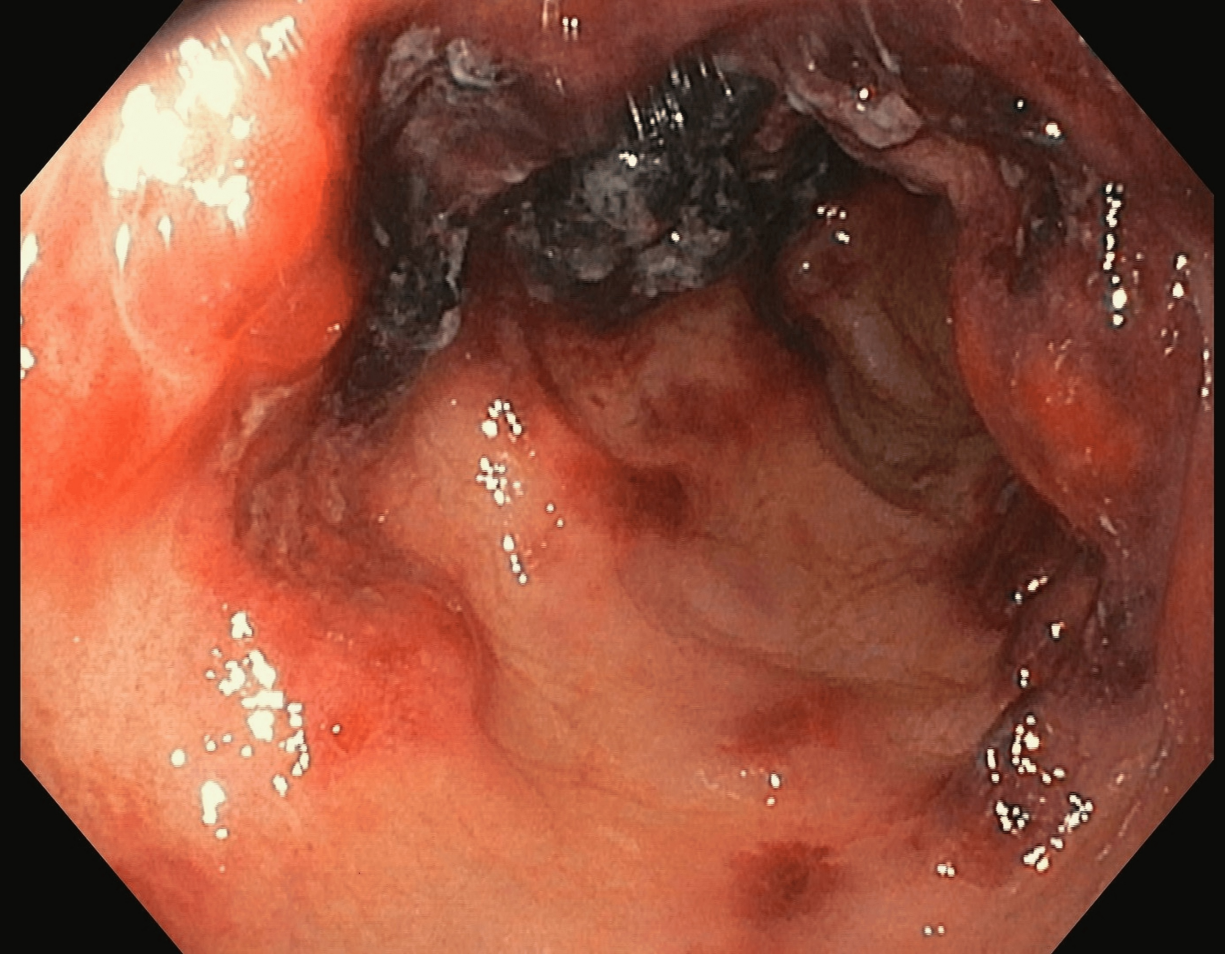
Colonoscopic Image Showing Multiple Erosions With Few Adherent Clots

In the MICU, fluid resuscitation was continued, and an arterial line was secured. Given the presence of bloody diarrhoea and diffuse abdominal pain, the medical gastroenterology team was consulted. A colonoscopy was done at the bedside by the medical gastroenterology team, showing grossly edematous intestinal mucosa with haemorrhage (Figures 4-5).

A colonoscopy revealed multiple erosions with a few adherent clots in both the large and small intestines. A biopsy was taken, considering the possibility of mesenteric ischemia or an opportunistic infection. Stool BioFire® FilmArray® (GI panel) (BioFire Diagnostics, LLC, Salt Lake City, USA) was sent to rule out infectious causes, which returned negative. Serologies for Herpes Simplex Virus (HSV) IgM and Cytomegalovirus (CMV) IgM were also negative. Biopsy was reported as non-specific colitis with mucosal haemorrhage, and there was no evidence of vasculitis. Based on these findings, she was diagnosed with haemorrhagic enterocolitis secondary to CPA therapy (Common Terminology Criteria for Adverse Events (CTCAE) version 5.0, Grade 4). CPA was discontinued. Her loose stool frequency decreased over time, and vasopressors were tapered and stopped. The acute kidney injury (AKI) started resolving, and hence she was shifted to the ward. In the ward, she was monitored for another week and was discharged in stable condition.

## Discussion

CPA is an anti-neoplastic agent when used in lower doses it causes immunosuppression. The drug decreases the secretion of interferon-gamma and interleukin-12 (IL-12) while increasing the secretion of T-helper 2 (Th2) cytokines like IL-4 and IL-10 in the CSF and peripheral blood. It has been used for autoimmune disorders and the management of post-transplant rejections. For Sjögren’s syndrome, the CPA infusion protocol has been successfully tried. Haemorrhagic cystitis and gonadal toxicity are two common side effects of CPA therapy. Cardiotoxicity, pulmonary toxicity and hepatotoxicity are a few rare side effects [13-16]. CPA-induced haemorrhagic enterocolitis is extremely rare. To the best of our knowledge, only three cases of severe enteritis and one case of haemorrhagic enterocolitis secondary to CPA therapy have been reported so far. The first case was reported by Yang et al. where a patient presented with watery diarrhoea post-CPA administration for microscopic polyangiitis [9]. Sato et al. reported the second case where granulomatosis with polyangiitis patient developed loose stools post-CPA therapy [10]. Abdominal CT showed diffuse wall thickening in small and large intestines, while colonoscopy showed erythematous, denuded mucosa. The third case was reported by Yoshida et al. where a patient developed similar complaints post-CPA therapy. CT showed similar features and the biopsy ruled out vasculitis [11]. Bujakowska et al. reported a case of haemorrhagic enterocolitis in a polymyositis patient treated with CPA, which resolved post-discontinuation of CPA and hydrocortisone enema [12].

Our patient was being treated with CPA infusion therapy for Sjögren’s syndrome, which included injection (Inj.) CPA 500 mg in 300 mL NS over three hours and Inj. mesna 200 mg pre- and post-CPA infusion to decrease complications. After the fifth cycle, she presented with bloody diarrhoea and vomiting. She underwent extensive evaluation, and infectious causes were ruled out with stool BioFire® FilmArray® (GI panel). CECT of the abdomen revealed diffuse intestinal wall thickening. The colonoscopy showed diffuse mucosal oedema with haemorrhage, multiple erosions, and a few adherent clots. The biopsy was negative for vasculitis, with no evidence of granulomatous inflammation, dysplasia, or malignancy. *Clostridium difficile* toxin, CMV IgM, and HSV IgM were also negative. Based on clinical features, imaging, laboratory tests, colonoscopic findings, and histopathology, she was diagnosed with haemorrhagic enterocolitis. CPA was discontinued, fluids and electrolytes were replaced, and steroids were continued. Vasopressors were gradually tapered and stopped, AKI resolved, and she showed clinical improvement. She was discharged on oral steroids.

The pathophysiology of drug-induced haemorrhagic enterocolitis remains unclear. CPA targets rapidly dividing tumour cells as well as healthy cells. Its impact on rapidly dividing intestinal stem cells, leading to extensive mucosal necrosis without subsequent regeneration, is a potential mechanism but requires further investigation. Viaud et al. reported that CPA altered intestinal microbiota, resulting in bacterial translocation in a mouse model [17]. However, additional case reports and studies are necessary to better understand the pathophysiology and identify risk factors for the development of drug-induced haemorrhagic enterocolitis.

## Conclusions

Even though extremely rare in the available literature, CPA-induced haemorrhagic enterocolitis can lead to fatal outcomes. Its diagnosis requires increased awareness among treating physicians and thorough evaluation, including abdominal CT, laboratory investigations, endoscopy, and biopsy. Discontinuation of the drug, along with supportive therapy, is the mainstay of treatment.
